# Dual Primary Gastric Adenocarcinoma and Diffuse Large B-Cell Lymphoma: A Unique Case With a Cascade of Rare Complications

**DOI:** 10.7759/cureus.94399

**Published:** 2025-10-12

**Authors:** Idan Grossmann, Harshavardhan Sanekommu, Natasha Campbell, Lee Peng, Shuvendu Sen

**Affiliations:** 1 Department of Internal Medicine, Hackensack Meridian Health Jersey Shore University Medical Center, Neptune City, USA; 2 Department of Gastroenterology and Hepatology, Hackensack Meridian Health Jersey Shore University Medical Center, Neptune City, USA

**Keywords:** diffuse large b-cell lymphoma, gastric adenocarcinoma, gastric outlet obstruction, gastrointestinal complications, synchronous primary tumors

## Abstract

Concurrent presentation of two primary gastrointestinal cancers is extremely rare. We describe an unusual presentation of a 69-year-old patient without a significant past medical history, who was diagnosed simultaneously with diffuse large B-cell lymphoma and gastric adenocarcinoma. This case is distinguished by the development of severe, rarely reported complications such as duodenocolonic fistula, bowel perforation, pulmonary embolism, and multi-organ failure. The combination of those complications underscores the exceptional complexity of management.

## Introduction

Primary gastrointestinal (GI) lymphoma is uncommon, comprising less than 5% of GI malignancies. Gastric adenocarcinoma, on the other hand, remains one of the most prevalent GI cancers worldwide. The synchronous occurrence of diffuse large B-cell lymphoma (DLBCL) and gastric adenocarcinoma in the same patient is exceedingly rare. Gastrointestinal lymphomas most commonly originate in the stomach, followed by the small intestine and colon, and are predominantly of the non-Hodgkin B-cell type, with DLBCL being the most frequent subtype. The estimated incidence of primary gastric lymphoma is approximately one to two cases per 100,000 individuals per year, while small intestinal lymphomas, including those of the duodenum, are even less common. In contrast, gastric adenocarcinoma accounts for 90-95% of all primary gastric malignancies and remains a leading cause of cancer-related mortality worldwide. Although both tumors can arise within the gastrointestinal tract, they differ substantially in their pathogenesis, biological behavior, and therapeutic approach. Understanding these distinctions is crucial, as the synchronous presentation of two distinct malignancies requires individualized management strategies that balance the timing and compatibility of surgery and chemotherapy to optimize patient outcomes [1,2].

Dual primary tumors pose significant therapeutic challenges due to their rarity, and the absence of standardized, concurrently effective treatment strategies can make clinical decision-making particularly complex. The management of synchronous gastric adenocarcinoma and DLBCL requires a multidisciplinary team approach that integrates different treatment modalities. While surgery is the standard of care for gastric adenocarcinoma, and chemotherapy (typically R-CHOP (rituximab, cyclophosphamide, doxorubicin, vincristine, and prednisone)) is the cornerstone for DLBCL, the timing, sequence, and compatibility of these interventions demand careful clinical judgment tailored to the individual patient's condition [3]. 

In this case, we present a 69-year-old patient who was diagnosed with dual advanced gastrointestinal malignancies complicated by gastric outlet obstruction, fistulous connections, and perforation. This case highlights the substantial clinical challenges involved in managing synchronous tumors, particularly when compounded by severe gastrointestinal complications.

## Case presentation

A 69-year-old male with a past medical history of inguinal hernia repair presented with one week of intractable nausea and vomiting. Two months earlier, he had experienced abdominal discomfort, distention, anorexia, and a 15-pound weight loss over four weeks. He denied fevers, chills, blood in the stool, a history of inflammatory bowel disease or colon cancer, smoking, or alcohol consumption. His occupational history included roofing with potential exposure to asbestos and chemicals throughout his career.

On admission, he was afebrile and hemodynamically stable, with mild abdominal tenderness. Laboratory studies revealed a white blood cell count of 13.3 K/μL, hemoglobin of 9.5 g/dL (baseline 11 g/dL), sodium of 133 mmol/L, and alkaline phosphatase of 657 U/L (Table 1).

**Table 1:** Laboratory results on admission AST: aspartate aminotransferase; ALT: alanine transaminase

Test	Patient Value	Reference Range
White blood cell count	13.3 ×10⁹/L	4.0 – 10.5 ×10⁹/L
Hemoglobin	9.5 g/dL	12.0 – 16.0 g/dL
Hematocrit	29%	36 – 46%
Platelets	210 ×10⁹/L	150 – 400 ×10⁹/L
Sodium	133 mmol/L	135 – 145 mmol/L
Potassium	4.1 mmol/L	3.5 – 5.0 mmol/L
Creatinine	1.0 mg/dL	0.6 – 1.2 mg/dL
Blood urea nitrogen	15 mg/dL	7 – 20 mg/dL
AST	30 U/L	10 – 40 U/L
ALT	38 U/L	7 – 56 U/L
Alkaline phosphatase	657 U/L	40 – 129 U/L
Total bilirubin	1.1 mg/dL	0.2 – 1.2 mg/dL
Albumin	3.2 g/dL	3.5 – 5.0 g/dL

**Figure 1:**
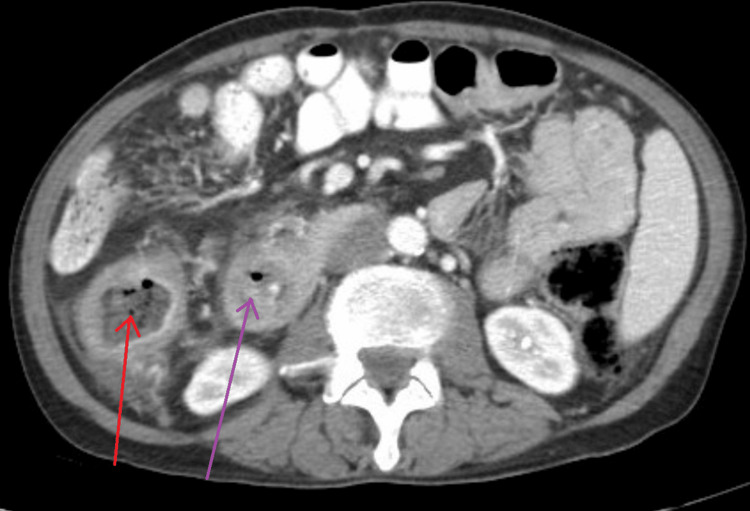
Abdominal CT demonstrating circumferential mural thickening of the ascending colon (red arrow) and duodenum (purple arrow)

**Figure 2:**
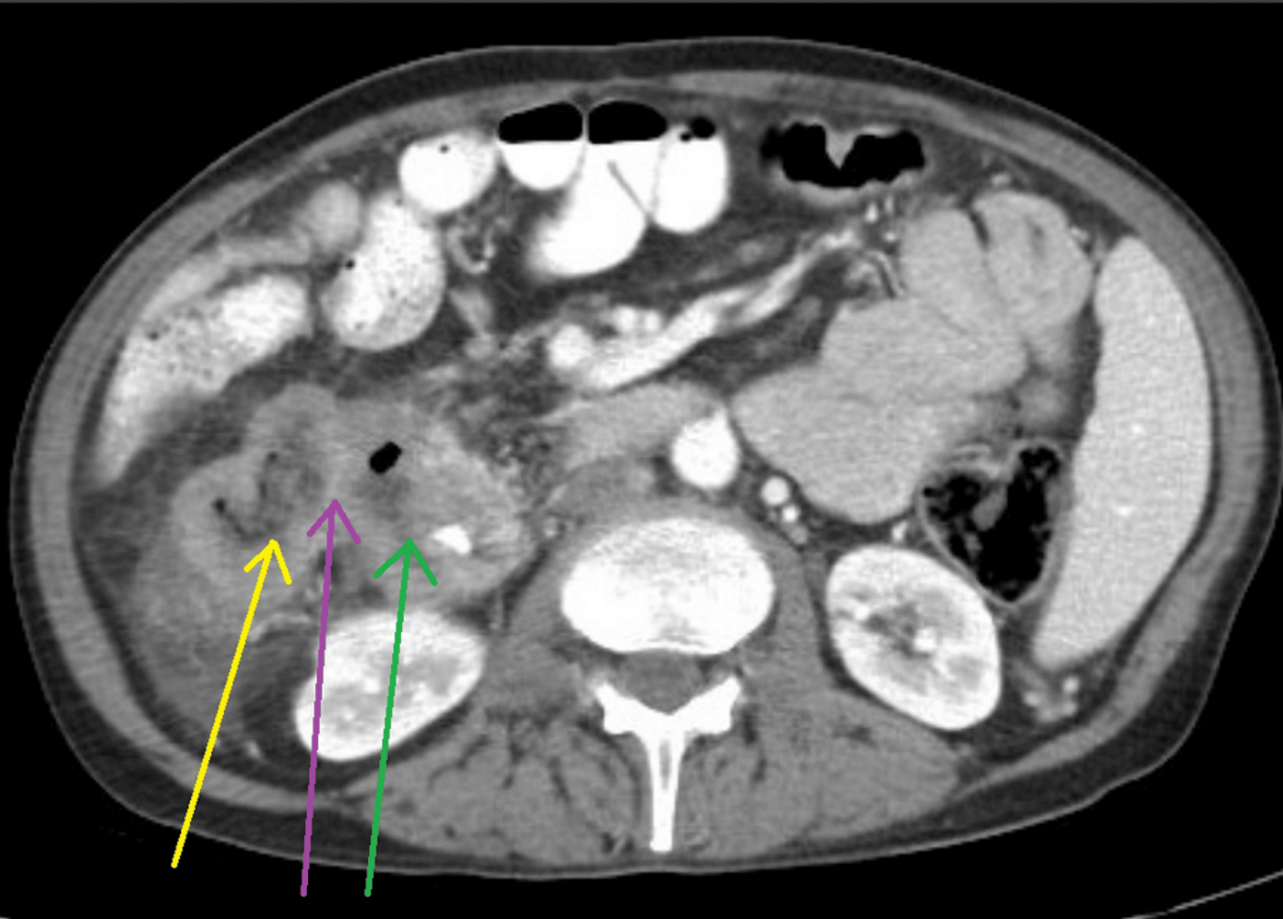
Abdominal CT image demonstrating a fistulous communication (purple arrow) between the duodenum (green arrow) and the adjacent ascending colon (yellow arrow)

**Figure 3:**
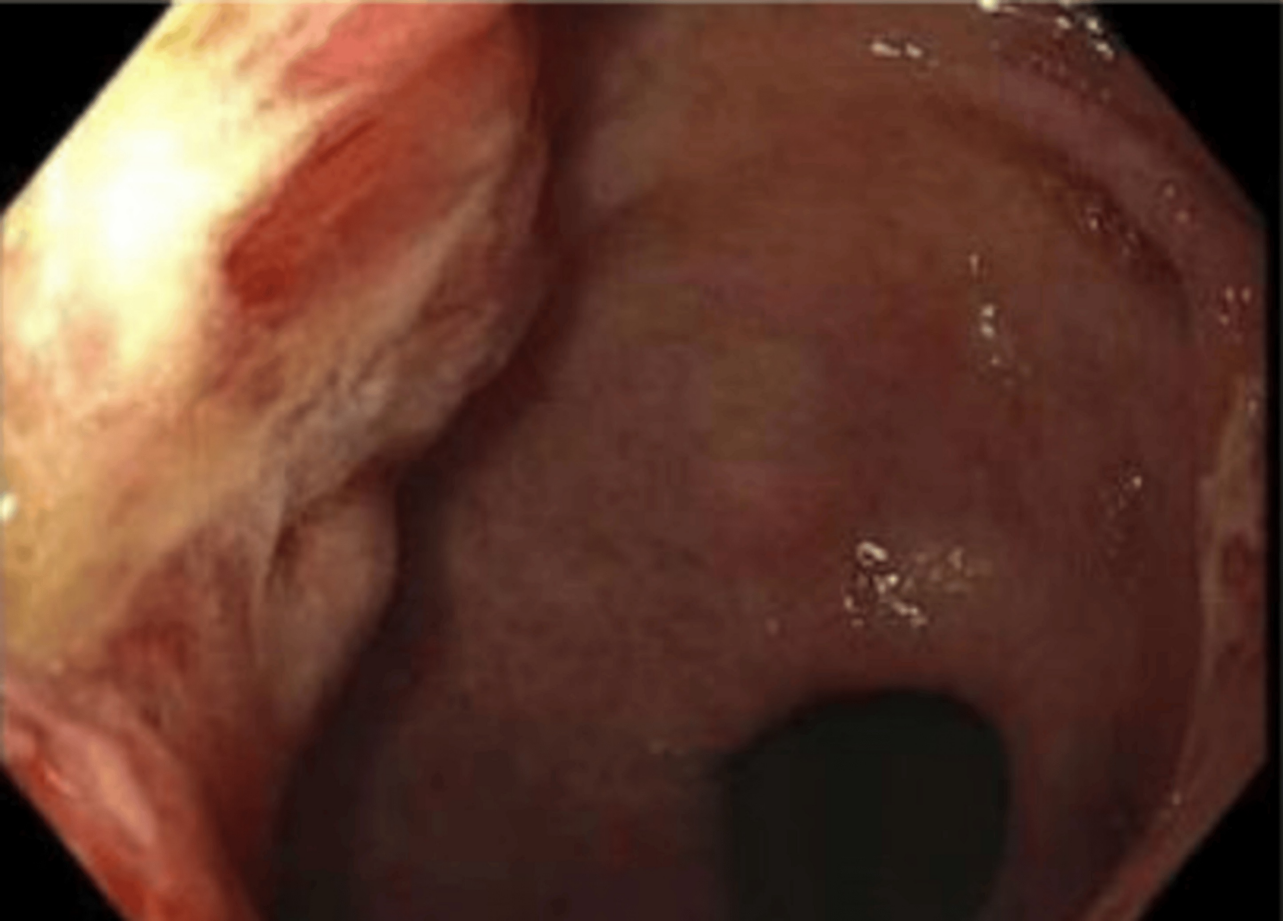
Endoscopic image showing a non-bleeding cratered mass in the gastric antrum.

**Figure 4:**
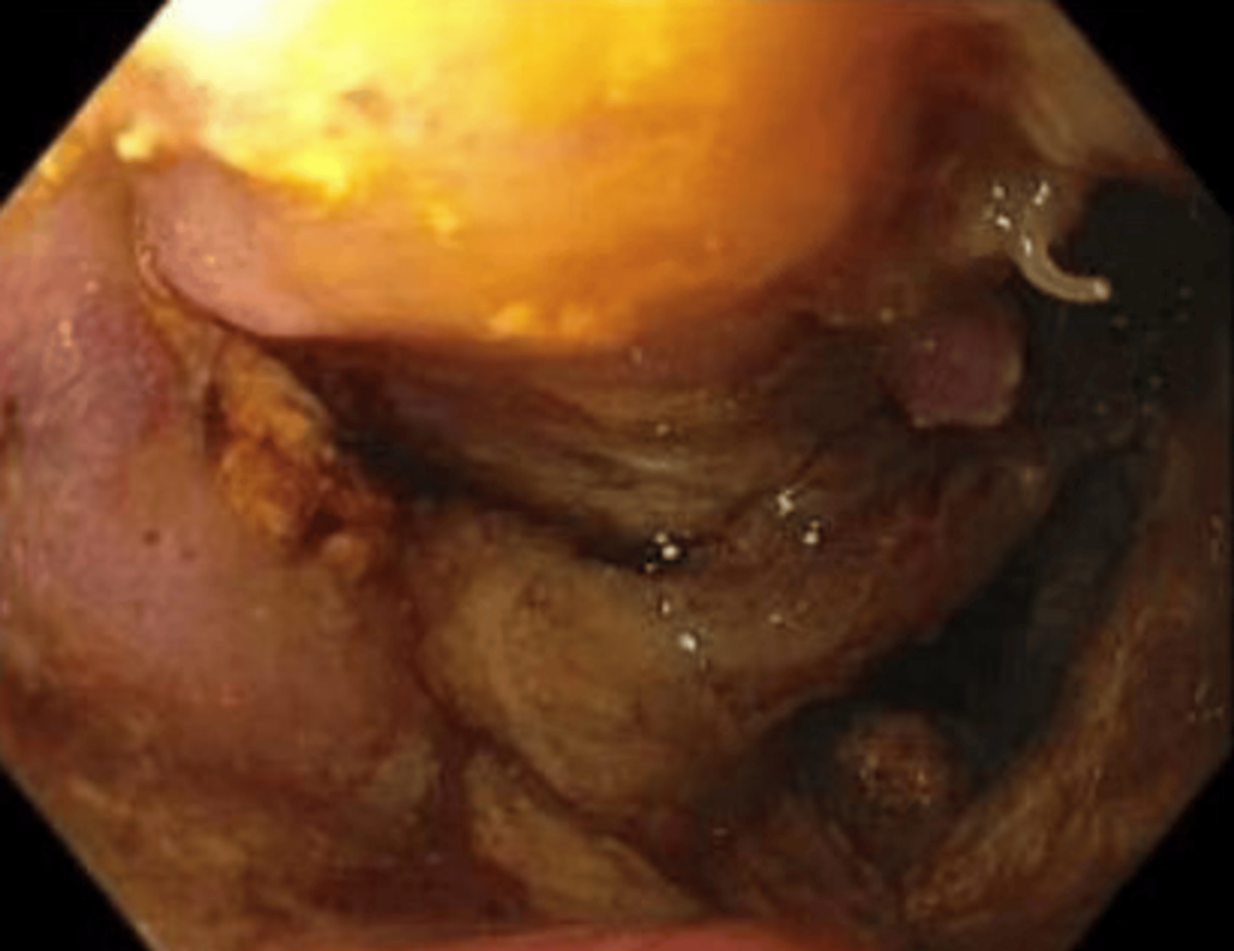
Endoscopic image demonstrating a large fungating and infiltrative mass in the third portion of the duodenum

A computed tomography (CT) scan of the abdomen and pelvis showed mural thickening of the duodenum and colonic thickening (Figure 1), as well as a fistula between the duodenum and ascending colon (Figure 2). Esophagogastroduodenoscopy revealed a 4 cm cratered gastric ulcer (Figure 3) and a large fungating duodenal mass causing gastric outlet obstruction (Figure 4), which was managed with nasogastric decompression.

Biopsies confirmed T3N2 poorly differentiated gastric adenocarcinoma with signet ring cells according to the American Joint Committee on Cancer (AJCC) eighth edition [4], based on clinical staging with N2 nodal involvement determined by imaging and endoscopic biopsy of regional lymph nodes, without prior lymphadenectomy, and DLBCL involving the duodenum with colonic fistula (WHO fifth edition [5]. These findings established the presence of two distinct primary malignancies: gastric adenocarcinoma and duodenal DLBCL.

Following confirmation of the diagnoses, given the high responsiveness of the duodenal lymphoma to systemic therapy and the high risk of immediate surgery due to obstruction, fistula, and friable bowel, the patient was started on R-CHOP chemotherapy. During treatment, the patient developed bilateral segmental pulmonary emboli. Although anticoagulation had initially been withheld due to the high risk of gastrointestinal bleeding, it was promptly initiated with therapeutic low-molecular-weight heparin upon confirmation of the pulmonary emboli.

The patient subsequently acutely decompensated with respiratory failure and bowel perforation, necessitating emergent surgery including right colectomy, partial Whipple procedure, and gastrectomy. Intraoperative findings revealed that the perforation resulted from extensive transmural infiltration, ischemic necrosis, and tissue breakdown caused by aggressive duodenal lymphoma, further complicated by the formation of a duodenocolic fistula, rather than any procedural or iatrogenic injury.

Postoperatively, the patient was admitted to the ICU in septic shock, requiring high-dose norepinephrine and vasopressin. Broad-spectrum antibiotics, initially including piperacillin-tazobactam and vancomycin, were tailored after intra-abdominal cultures grew *Escherichia coli* and *Bacteroides fragilis*. Despite maximal support with vasopressors, mechanical ventilation, renal replacement therapy, and transfusions, the patient developed refractory multiorgan failure. After shared decision-making with the family regarding the poor prognosis, care was transitioned to hospice. 

## Discussion

This case describes an exceptionally uncommon presentation of two distinct primary gastrointestinal cancers that were seldom reported to coexist simultaneously. The literature has documented 56 cases of gastric cancer and lymphoma occurring simultaneously since the first case was reported in 1855 [1,2].

The clinical presentation of synchronous gastric adenocarcinoma and DLBCL is highly variable and can lead to markedly different clinical courses and outcomes. Carboni et al. described a patient with synchronous DLBCL and gastric adenocarcinoma who exhibited progressive weakness, persistent dyspepsia, and significant weight loss [2], while Hao et al. described a patient who presented with a primary complaint of a choking feeling after eating, associated with epigastric abdominal pain and discomfort [6]. Both patients were treated successfully with chemotherapy without major complications. However, the clinical course can be severely complicated. Sakr et al. reported a patient who experienced epigastric pain and weight loss and subsequently developed multiorgan failure [7]. Our patient, who initially presented with vague, nonspecific symptoms, progressed to a severe and rarely reported cascade of complications, including duodenocolonic fistula formation and bowel perforation requiring emergent surgery. It demonstrates the importance of a high level of suspicion in the setting of non-specific symptoms, as delays might lead to severe, irreversible complications.

Our case emphasizes the therapeutic dilemma involved in approaching a patient with dual gastrointestinal malignancy. Due to the infrequency of dual primary gastrointestinal cancer, the recommended management of this scenario is not well established and requires tailored clinical decision-making. In our case, the complication of the obstructing duodenal lymphoma required us to urgently address and treat this primary cancer first before treating the gastrointestinal adenocarcinoma.

Despite an initial plan to prioritize lymphoma treatment with R-CHOP and defer surgery, the patient rapidly decompensated due to bowel perforation and required extensive surgical intervention. The perforation was attributed to disease-related transmural necrosis and fistulous disruption rather than procedural complications, highlighting the aggressive nature of synchronous gastrointestinal malignancies. It further emphasizes the importance of early diagnosis to reduce the risk of complications that may compromise overall treatment success. Furthermore, it illustrates the high morbidity burden associated with dual pathology, demonstrated by the multiple complications of pulmonary embolism, intra-abdominal sepsis, and multi-organ failure. This case meaningfully contributes to the limited literature on coexisting primary gastrointestinal malignancies, a phenomenon that has been rarely documented. It highlights the significant diagnostic and therapeutic challenges such presentations pose, often requiring comprehensive clinical judgment and multidisciplinary decision-making. Moreover, it underscores the necessity of a high index of suspicion when evaluating patients with unexplained weight loss and abdominal complaints, and the need for additional case reports to expand our understanding and help guide evidence-based management strategies.

## Conclusions

The coexistence of gastric adenocarcinoma and diffuse large B-cell lymphoma represents an exceptionally rare clinical scenario with no standardized management approach. This case illustrates how the simultaneous occurrence of two aggressive malignancies, compounded by severe complications, can lead to rapid clinical deterioration despite appropriate interventions. Early recognition, multidisciplinary collaboration, and continued reporting of such cases are essential to improve understanding and guide evidence-based strategies for future management.
